# Toward Chemical Accuracy Using the Jastrow Correlated Antisymmetrized Geminal Power *Ansatz*

**DOI:** 10.1021/acs.jctc.2c01141

**Published:** 2023-04-04

**Authors:** Abhishek Raghav, Ryo Maezono, Kenta Hongo, Sandro Sorella, Kousuke Nakano

**Affiliations:** † International School for Advanced Studies (SISSA), Via Bonomea 265, 34136 Trieste, Italy; ‡ School of Information Science, Japan Advanced Institute of Science and Technology (JAIST), Asahidai 1-1, Nomi, Ishikawa 923-1292, Japan; ¶ Research Center for Advanced Computing Infrastructure, Japan Advanced Institute of Science and Technology (JAIST), Asahidai 1-1, Nomi, Ishikawa 923-1292, Japan; § Research and Services Division of Materials Data and Integrated System, National Institute for Materials Science (NIMS), Tsukuba, Ibaraki 305-0047, Japan

## Abstract

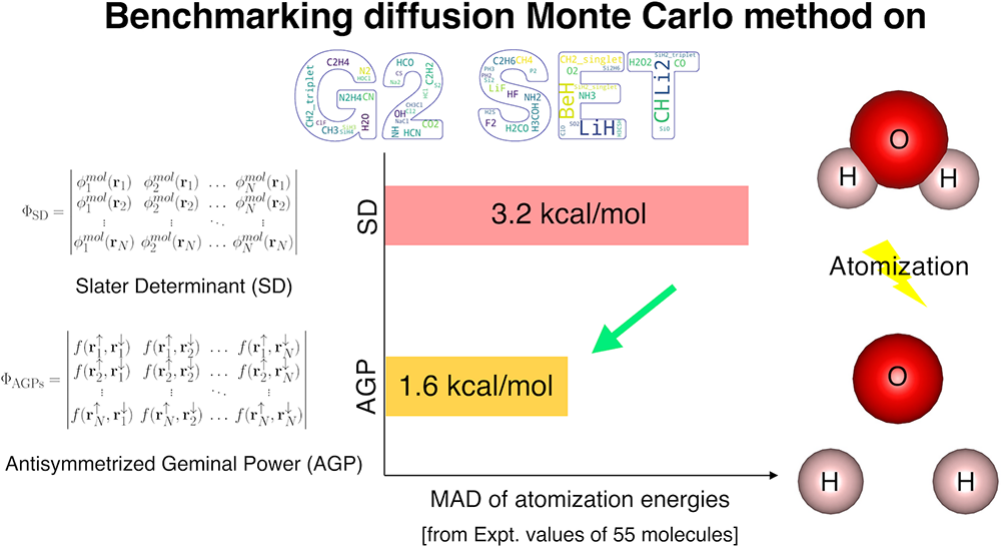

Herein, we report accurate atomization energy calculations for 55 molecules in the Gaussian-2 (G2) set using lattice regularized diffusion Monte Carlo (LRDMC). We compare the Jastrow–Slater determinant *ansatz* with a more flexible JsAGPs (Jastrow correlated antisymmetrized geminal power with singlet correlation) *ansatz*. AGPs is built from pairing functions, which explicitly include pairwise correlations among electrons, and hence, this *ansatz* is expected to be more efficient in recovering the correlation energy. The AGPs wave functions are first optimized at the variational Monte Carlo (VMC) level, which includes both the Jastrow factor and the nodal surface optimization. This is followed by the LRDMC projection of the *ansatz*. Remarkably, for many molecules, the LRDMC atomization energies obtained using the JsAGPs *ansatz* reach chemical accuracy (∼1 kcal/mol), and for most other molecules, the atomization energies are accurate within ∼5 kcal/mol. We obtained a mean absolute deviation of 1.6 kcal/mol with JsAGPs and 3.2 kcal/mol with JDFT (**J**astrow factor + Slater determinant with **DFT** orbitals) *ansatzes*. This work shows the effectiveness of the flexible AGPs *ansatz* for atomization energy calculations and electronic structure simulations in general.

## 1 Introduction

*Ab initio* quantum Monte Carlo (QMC) techniques,^1^ such as variational Monte Carlo (VMC) and diffusion Monte Carlo (DMC), have been used to compute accurate many-body wave functions (WF) for atomic, molecular, simple crystal, and even complex electronic systems.^2−4^ Benchmark results are of great interest for QMC methods because these methods have the capability to match the accuracy of quantum chemical methods and also scale more favorably than several wave function based methods.^1^ A WF-based quantum chemical method, such as the coupled-cluster with single and double and perturbative triple excitations (CCSD(T)), scales as *O*(*N*^7^), where *N* is the number of electrons.^5,6^ In contrast, DMC at most scales as *O*(*N*^4^) and is closer to the computational scaling of *O*(*N*^3^) (for *N* < 1000–2000)^7^ of the more traditional mean-field methods like DFT. Since there is a large prefactor involved in the DMC method, the WF-based method is often more favorable for smaller systems than DMC. However, as the system size increases, the scaling becomes the dominating factor in their computational costs. It limits the application of the *conventional* CCSD(T) to systems of up to ∼80–90 electrons,^8^ while recent implementations extend this limit further, e.g., up to ∼300 electrons for molecules^9^ and ∼100 electrons for solids.^10^ On the other hand, for QMC methods, system sizes up to ∼2000 electrons are accessible, which makes them invaluable for condensed matter calculations.^7^ Thus, making QMC methods more accurate and efficient using various approaches (like using the more flexible WF *ansatz*) is an active area of research.

A widely used set of benchmark data is the atomization energies of the Gaussian-2 (G2) set of molecules.^11^ It contains 55 small molecules composed of elements from the first, second, and third rows of the periodic table. Accurate experimental values for the atomization energies of the G2 set of molecules are available, which makes it very attractive for benchmarking. Another way to benchmark is to compare the computed total energies with the estimated exact energies. However, to evaluate the error cancellation, it is important to benchmark the atomization energies.

The G2 set benchmarks have been used to test several state-of-the-art *ab initio* computational methods. Chemical accuracy (deviation of 1 kcal/mol from experimental estimates of atomization energies) has often been used as the target accuracy. The benchmarking relative to molecular atomization energies of DFT methods in previous studies resulted in a relatively large mean absolute deviation (MAD) value of ∼40 kcal/mol for local density approximation (LDA) and ∼2.5 kcal/mol for the hybrid B3LYP functional.^12^ In another study, B3LYP on an extended G2 set gave an MAD of 3.11 kcal/mol,^13^ with a maximum deviation of ∼20 kcal/mol. This large deviation suggests that DFT methods are not systematically accurate, and there might be significant variation in the accuracy across various systems. Efforts have been made to improve the DFT estimates by determining correction factors from fitting to experimental data;^14^ however, these reduce the prediction ability of the theory. Coupled cluster theory based methods such as CCSD(T) have been largely regarded as the “gold standard” for accuracy in quantum chemistry. Several CCSD(T) studies have shown that the method can achieve subchemical accuracy if large enough basis sets and multiple corrections are used.^5,15−18^

QMC methods lie on the sweet spot of accuracy and computational cost. They are far more accurate than mean-field methods (no inherent approximation like the XC functional) and have a more favorable scaling than the WF-based quantum chemical methods. CCSD(T)/aug-cc-pVQZ gave an MAD of 2.8 kcal/mol for the G2 set atomization energies. When extrapolated to the complete basis set limit, the MAD value was reduced to 1.3 kcal/mol.^19^ Several FN-DMC benchmark tests (using single SD *ansatz*) for the G2 set atomization energies have obtained MAD values close to 3 kcal/mol. For instance, Nemec et al.^20^ used all-electron FN DMC on Slater determinant (SD) WF to obtain the atomization energies of the G2 set to an MAD of 3.2 kcal/mol. Similar accuracy in atomization energies was also obtained previously in DMC pseudopotential calculations.^21^ However, these simple DMC approaches (i.e., Jastrow + SD) have not been able to achieve chemical accuracy or subchemical accuracy due to the residual FN errors.

In this paper, we present FN DMC benchmark results using the so-called AGPs (antisymmetrized geminal power with singlet correlation) *ansatz*(22) along with a Jastrow factor (JF). Combined with VMC optimization, the more flexible AGPs *ansatz* leads to improved nodal surfaces. The main outcome of this work is that the combination of a more flexible *ansatz* (AGPs) and nodal surface optimization leads to a much better quality many-body WF, which in turn leads to better DMC energies. This is very important because, AGPs (even though being multiconfigurational in nature^4^) in practice is as efficient as a single determinant *ansatz* and thus can be extended to much larger systems, even within the computationally demanding QMC methods. QMC also provides an added advantage of the near ideal parallel scaling of QMC algorithms.^4^

## 2 Computational Details

The TURBORVB(4) QMC package was used for all calculations. It employs resonating valence bond (RVB^23^) WF and allows one to choose a more flexible *ansatz* than the SD *ansatz* and includes correlation effects beyond the standard SD.

### 2.1 Wave Functions

The choice of the WF *ansatz* plays an important role in determining the accuracy and the computational cost of QMC calculations. A many-body WF *ansatz* can be written as the following product
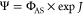
1where exp *J* is the JF, and Φ_AS_ is the antisymmetric part that satisfies the antisymmetry condition for fermions. Generally, a single SD is used for the antisymmetric part in QMC calculations. SD is simply an antisymmetrized product of single particle electron orbitals and does not include any electron correlation by itself.

#### 2.1.1 Jastrow Factor

The Jastrow factor is a multiplier term which improves the quality of a many-body WF by providing a significant portion (≈70%) of correlation energy and is necessary for fulfilling Kato’s cusp conditions.^24^ The JF used here comprises three terms: one-body, two-body, and three/four-body terms (*J* = *J*_1_ + *J*_2_ + *J*_3/4_). The one-body term is necessary to satisfy the electron–ion cusp condition. A separate one-body term is used for each element present in the molecule. It consists of the so-called homogeneous part
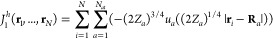
2and the so-called inhomogeneous part
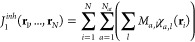
3where **r**_*i*_ denotes the electron coordinates, **R**_*a*_ represents the atomic positions, *Z*_*a*_ denotes the corresponding atomic numbers, *N*_*at*_ is the number of nuclei, χ_*a*,*l*_ represents a Gaussian-type atomic orbital *l* centered over atom *a*, and *M*_*a*,*l*_ denotes the corresponding variational parameters. The function *u*_*a*_ is defined as
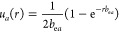
4where *b*_e*a*_ is a variational parameter that depends on each nucleus *a*. The homogeneous one-body term was carefully optimized at the DFT level before final optimization at the VMC level. The two-body term is necessary to satisfy the electron–electron cusp condition and is optimized at the VMC level
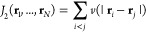
5The function *v* has the following form
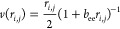
6where *r*_*i*,*j*_ = |**r**_*i*_ – **r**_*j*_|, and *b*_ee_ is a single variational parameter. The three/four-body Jastrow term is defined as
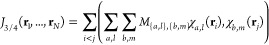
7where *M*_{*a*,*l*},{*b*,*m*}_ represents the variational parameters, and *l* and *m* indicate orbitals centered on atomic sites *a* and *b*, respectively. In the current work, the four-body Jastrow term (i.e., *a* ≠ *b*) was not used.

#### 2.1.2 Antisymmetrized Geminal Power (AGP)

One way to improve the description of electron correlations and move beyond the SD is to explicitly include pairwise correlation among electrons. This fairly general and flexible *ansatz* is called the Pfaffian WF.^25^ The Pfaffian WF is constructed from pairing functions (known as geminals). When only the singlet electron pairing terms are considered, we get the AGPs *ansatz*.^22^ A generic pairing function for the AGPs, *f*(**r**_*i*_, **r**_*j*_), can be written as
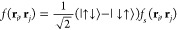
8For a simpler unpolarized case, where the number of electrons *N* is even and *N*_*↑*_ = *N*_*↓*_, all possible combinations of singlet pairs can be written in the form of a matrix:
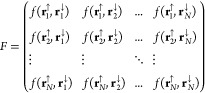
9The determinant of matrix *F* gives the AGPs WF:
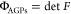
10In the case of an open-shell chemical system (*N*_*↑*_ ≠ *N*_*↓*_), matrix *F* consisting of singlet terms only would become rectangular matrix. To convert matrix *F* into a square matrix, additional unpaired molecular orbitals (Θ_*i*_(**r**)) are added to matrix *F*. Suppose *N*_*↑*_ > *N*_*↓*_, *N*_*↑*_ – *N*_*↓*_ unpaired spin-up molecular orbitals are added to matrix *F*:
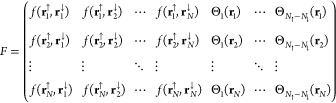
11det *F* now gives the antisymmetric WF.

The singlet electron pairing terms are represented by
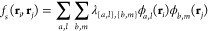
12where ϕ_*a*_ and ϕ_*b*_ represent atomic orbitals centered on atoms *a* and *b*, respectively, and the indices *l* and *m* indicate different orbitals centered on atoms *a* and *b*, respectively. The elements of matrix λ are the coefficients or the variational parameters of the WF. An important advantage of the AGPs *ansatz* is that it is equivalent to a linear combination of SDs (or multideterminants) while maintaining the computational cost of a single determinant *ansatz*.^4^ Hence, this flexible *ansatz* (greater variational freedom) could be an effective way to improve the quality of the many-body WF.

### 2.2 Computational Workflow

The equilibrium geometries of the G2 set molecules were taken from previous benchmark studies^13,17,26^ (see Table S2 in the Supporting Information). The pairing function was expanded using the triple-ζ atomic basis sets obtained from the Basis Set Exchange library.^27^ Larger exponents greater than 8*Z*^2^ (where *Z* is the atomic number) were removed from the basis set to avoid numerical instabilities. The large exponent orbitals cut from the basis set are implicitly included by utilizing the one-body Jastrow term.^28,29^ The basis sets used for the determinant and Jastrow expansion are listed in Table. 1. The same basis sets were used for the VMC and LRDMC calculations.

**Table 1:** Basis set orbitals used for the determinant and Jastrow expansion

element	det. basis	jas. basis
H	4s, 2p, 1d	4s, 2p
Li	8s, 5p, 2d, 1f	8s, 5p, 2d
C	8s, 5p, 2d, 1f	8s, 5p, 2d
N	8s, 5p, 2d, 1f	8s, 5p, 2d
O	7s, 5p, 2d, 1f	7s, 5p, 2d
F	7s, 5p, 2d, 1f	7s, 5p, 2d
Na	11s, 10p, 2d, 1f	11s, 10p, 2d
Si	11s, 9p, 2d, 1f	11s, 9p, 2d
P	11s, 9p, 2d, 1f	11s, 9p, 2d
S	11s, 9p, 2d, 1f	11s, 9p, 2d
Cl	11s, 9p, 2d, 1f	11s, 9p, 2d

The trial WF for the Jastrow single determinant *ansatz* was obtained from DFT calculations using the TURBORVB DFT module. To improve the efficiency of the DFT calculations, we utilized the double-grid DFT algorithm, which used a finer DFT mesh when in the vicinity of the nuclei.^29^ For the JDFT (JF + SD with DFT orbitals) *ansatz*, the JF was optimized at the VMC level, which was followed by the LRDMC projection. In LRDMC, instead of the conventional time discretization^30^ of the continuous Hamiltonian, the regularization of the original Hamiltonian is done over the lattice with a step size *a*, such that  for *a* → 0.^31−33^ Further details on the VMC and LRDMC algorithms can be found in refs (34−38). The target error bar for the DMC and VMC energies was taken as ≈0.3 mHa. For the VMC optimization, we used the linear^39−41^ and stochastic reconfiguration methods.^42,43^ The JDFT WF *ansatz* was then converted into JsAGPs. No information loss occurs during this conversion because we are rewriting the SD *ansatz* into a more flexible AGPs *ansatz*, and maximum overlap between the two WFs is ensured. The JsAGPs was then optimized at the VMC level, including both JF and nodal surface optimization. This was followed by the LRDMC projection and extrapolation to zero lattice space. The complexity of VMC optimization can be roughly estimated by the number of variational parameters to be optimized. For instance, the number of variational parameters used for a simple system like BeH were 306 and 735 for JDFT and JsAGPs *ansatzes*, respectively. For a complex system like Si_2_H_6_, the number of variational parameters optimized were 872 and 6422 for JDFT and JsAGPs *ansatzes*, respectively. The variational energy, *E*[α], as well as the maximum value of the signal-to-noise ratio for forces (termed as devmax) was monitored
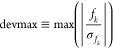
13where *f*_*k*_ and  denote force and the corresponding error bar, respectively. It has been observed that when devmax stabilizes to values < ∼4, an energy minimum with repect to the variational parameters is being approached.^4^ The same criteria was used (in addition to checking a general convergence of energy and one-/two-body Jastrow parameters) in general to decide when to stop VMC optimization. For instance, for Si_2_H_6_ VMC optimization was carried out for 4000 optimization steps using the linear method. For most of the molecules considered, the linear method was used for VMC optimization. For SiH_3_, C_2_H_2_, CH_2_ (triplet), and CH_3_, the stochastic method was used, requiring ∼15000–20000 optimization steps.

The majority of the calculations was performed on the supercomputer Fugaku using 2304 CPU cores distributed across 48 nodes. To improve the efficiency of the calculations, we used TURBOGENIUS, a python-based wrapper for TURBORVB, which is useful in performing high-throughput calculations.^4^

## 3 Results

### 3.1 JsAGPs for the N_2_ Molecule

To validate the methodology and verify whether the basis sets used were good enough to approach chemical accuracy, the energies of the N_2_ molecule and N atom were compared with previous benchmark tests and experimental values (see Table 2).

**Table 2:** Nitrogen Energies

	method	basis set	atom (Ha)	molecule (Ha)	atomization energy (eV)
	JDFT-VMC	cc-pVTZ	–54.5543(2)	–109.4522(3)	9.35(1)
	JDFT-DMC	cc-pVTZ	–54.5765(3)	–109.5068(3)	9.61(2)
	JsAGPs-VMC	cc-pVTZ	–54.5614(1)	–109.4702(5)	9.45(1)
current work (TURBORVB)	JsAGPs-DMC	cc-pVTZ	–54.5785(4)	–109.5165(3)	9.92(1)
	JHF-DMC^a^	QZ4P	–54.5765(2)	–109.5065(4)	9.61(2)
	CCSD(T)^b^	–	–	–	9.85(1)
	Fermi net^c^	–	–54.58882(6)	–109.5388(1)	9.828(5)
	JSD-DMC pseudopotential^d^	cc-pV5Z	–	–	9.573(4)
	estimated exact^e^	–	–54.5892	–109.5427	9.91
previous reports	experimental^f^	–	–	–	9.91

a Reference (20).

b Reference (17).

c Reference (44).

d Reference (45).

e Reference (46).

f Reference (47).

The JsAGPs-DMC atomization energy shows excellent agreement with the experimental value.^47^ It is better than the value computed using CCSD(T)^17^ and the value computed recently using a neural network based *ansatz* (called the Fermi net) .^44^ The correlation energies recovered for the N_2_ molecule at the JsAGPs-DMC level and the JDFT-DMC level were ≈95% and ≈93%, respectively. At the VMC level, for JsAGPs, ≈86% percent correlation energy was recovered. The computed JDFT-DMC energies are in excellent agreement with the ones computed by Nemec et al.^20^ Clearly, in the case of nitrogen, a triple-ζ basis set for orbital and Jastrow expansion was good enough. The best total (closest to the estimated exact) energies are the ones computed by Pfau et al. using Fermi net.^44^ The JsAGPs atomization energy, however, is more accurate than that obtained by Pfau et al. This shows that the JsAGPs *ansatz* allows remarkable cancellation of errors when the difference between the molecular and atomic energies is computed. Interestingly, the N_2_ atomization energy computed using the Jastrow Slater determinant (JSD) *ansatz* by Petruzielo et al.^45^ could not approach CCSD(T) level accuracy. Hence, for the JSD *ansatz*, Jastrow and nodal surface optimizations are not sufficient for improving the quality of the WF *ansatz*. Thus, the standard JSD might be inadequate for this purpose.

### 3.2 Application to the G2 Set

**Figure 1:**
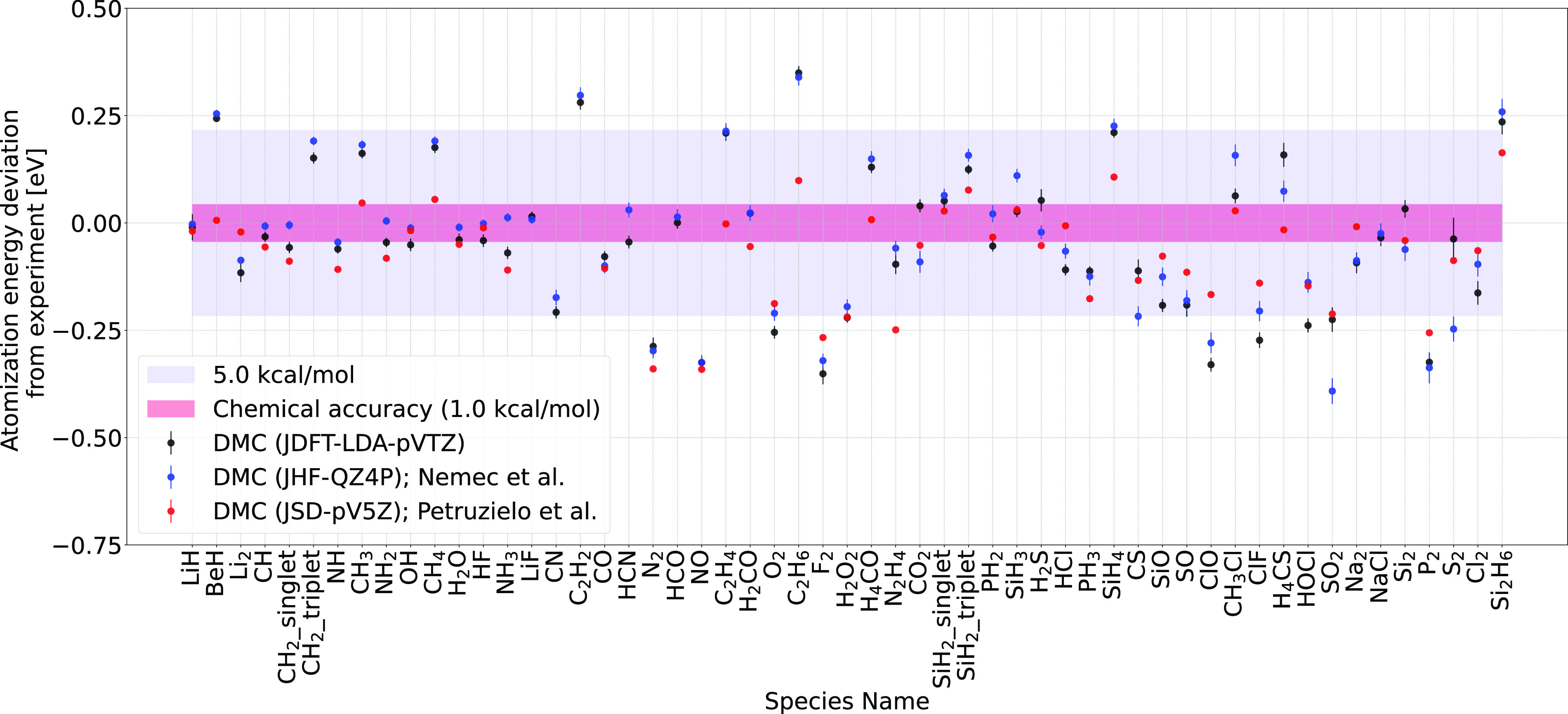
Deviation of the DMC atomization energies from the experimentally obtained values for the JDFT *ansatz* with the triple-ζ basis set. Zero point energies and relativistic + spin orbit were corrected before computing the deviations between the DMC and experimental values.^20^ Values obtained by Nemec et al.^20^ (JHF-QZ4P) and Petruzielo et al.^45^ (JSD-5z) are also plotted for comparison. The MAD for the JDFT *ansatz* is ≈3.2 kcal/mol, and the MAD values obtained by Nemec et al. and Petruzielo et al. are 3.13 and 2.1 kcal/mol, respectively.

Our JDFT-DMC atomization energies (Figure 1) were first compared with the ones obtained by Nemec et al.^20^ (Jastrow Hartee Fock (JHF)-DMC, QZ4P STO basis set) and Petruzielo et al.^45^ (JSD-DMC, 5z basis set). The results obtained for the JDFT-DMC atomization energies are in good agreement with the ones obtained by Nemec et al. Most atomization energies obtained using the JDFT *ansatz* were within a deviation of ±0.25 eV (±3.0 kcal/mol) from the experimental values although very few were in the chemical accuracy range. The JDFT atomization energies had an MAD of ≈3.2 kcal/mol, which is quite close to the value of ≈3.13 kcal/mol reported by Nemec et al. An MAD of 2.9 kcal/mol was reported in another FN DMC (atomic cores treated with pseudopotentials) G2 set benchmark by Grossman.^21^ This overall agreement with previous benchmark tests points out that FN DMC provides “near chemical accuracy”, and the primary sources of error are the fixed (unoptimized) nodes. There could be other sources of error, such as the basis set used for orbital expansion. However, it can be ruled out based on the fact that Nemec et al. used a larger basis set (Qz) than that used in the current study. Nonetheless, the errors in the atomization energies are quite similar. Comparison of the JDFT atomization energies with the JSD atomization energies (obtained by Petruzielo et al.) shows that optimizing the nodal surfaces improves the DMC atomization energy estimates over the ones obtained using DFT or mean-field nodal surfaces.

**Figure 2:**
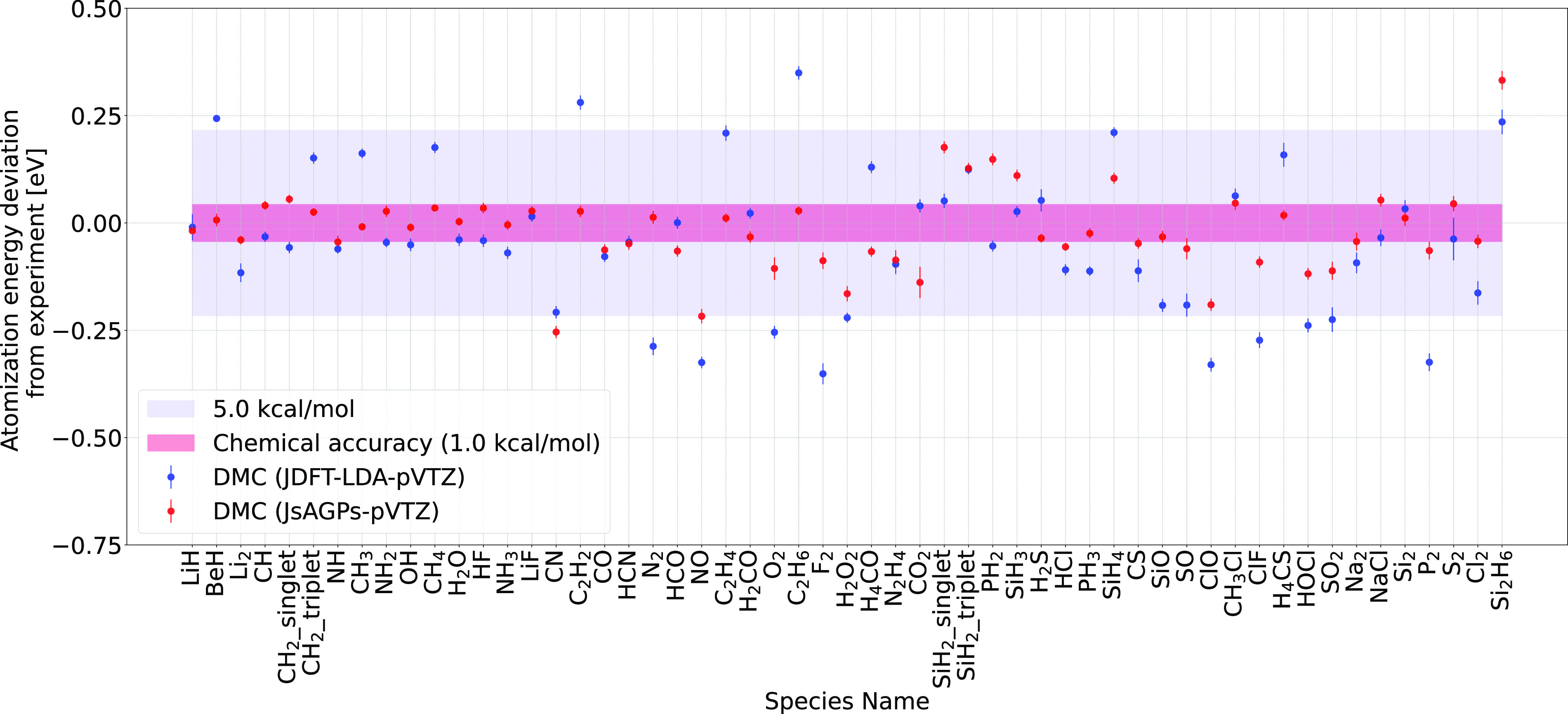
Deviation of the DMC atomization energies from the experimentally obtained values for the JDFT and JsAGPs *ansatzes*. The error bars are shown within the markers. The MAD from the experiment for the JDFT and JsAGPs *ansatzes* is ≈3.2 kcal/mol and ≈1.6 kcal/mol, respectively. Zero point energies and relativistic + spin orbit were corrected before computing the deviations between the DMC and experimental values.^20^

**Figure 3:**
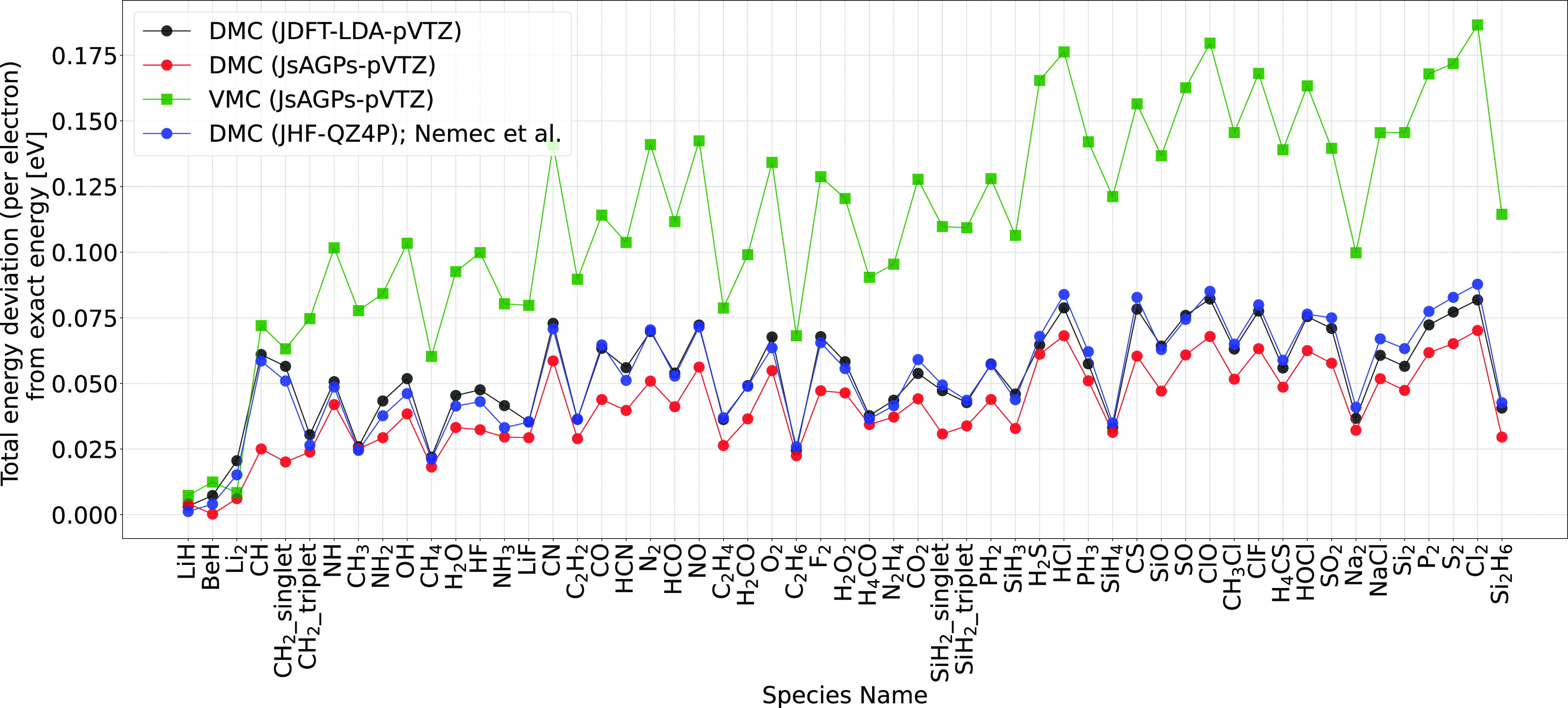
Deviation of the VMC and DMC total energies from the estimated exact energies. Values obtained by Nemec et al.^20^ are plotted for comparison.

**Figure 4:**
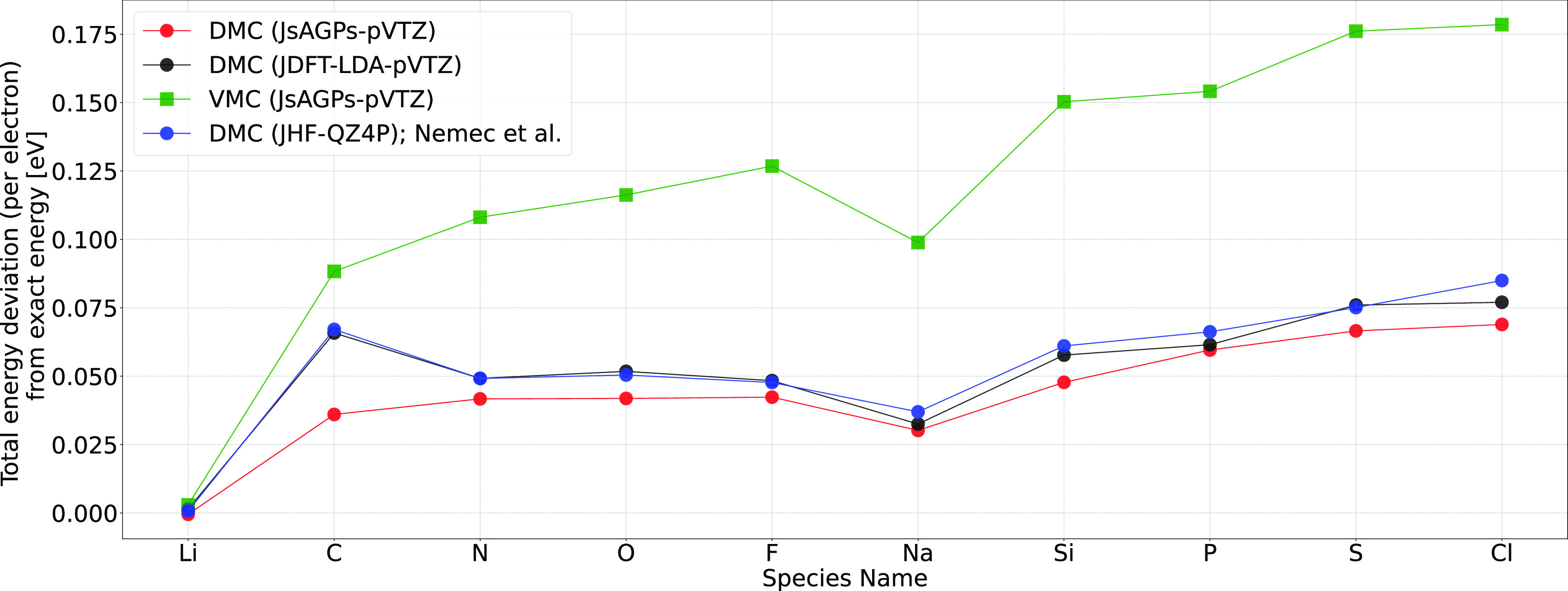
Deviation of the VMC and DMC total energies of atoms from the estimated exact energy. Values obtained by Nemec et al.^20^ are plotted for comparison.

AGPs, which is a more flexible *ansatz*, allows for better nodal surfaces. Figure 2 shows a comparison of the DMC atomization energy between the JDFT and the more flexible JsAGPs *ansatz*. Total energies are shown in Figure 3 and Figure 4. Clearly, the best variational energies were obtained when LRDMC was applied to the JsAGPs *ansatz* both for the atoms and the molecules. This means that the nodal surfaces of JsAGPs WF are better than those of JDFT, and hence, considerably more correlation energy is recovered. For example, in the case of atoms, an average of 95.6% correlation energy was recovered at the DMC level for the JsAGPs WF, and 93.7% correlation energy was recovered using the JDFT. In the case of atomization energies, JsAGPs is clearly better than JDFT. The MAD using the JsAGPs and JDFT *ansatzes* was 1.6 and 3.2 kcal/mol, respectively, demonstrating a clear superiority of the JsAGPs. For almost all the molecules, the error was within 5.0 kcal/mol, and chemical accuracy was achieved for 26 molecules in the G2 set. These results are not only better than the ones obtained using JDFT (with nodal surface from DFT) but also better than the case wherein the SD nodal surfaces were optimized by Petruzielo et al.^45^ The better atomization energies indicate that JsAGPs not only provides better variational energies but also improves error cancellation.

## 4 Discussion

It is interesting to note that for a few molecules (e.g., Si_2_H_6_, CO_2_), the JsAGPs atomization energies are worse than their JDFT counterparts. It turns out that although AGPs leads to better nodal surfaces (and lower DMC energies) than JDFT for all molecules and atoms, the quality of nodal surface depends upon the chemical structure of molecules and atoms, and hence, the error cancellation is not always predictably better.

In this study, we tried to reduce the FN error by optimizing the nodal surfaces at the variational level with the single-determinant *ansatz* (JsAGPs). It is important to compare our QMC results with those obtained with the multideterminant *ansatz* that is promising in terms of accuracy. Petruzielo et al.^45^ showed that DMC with the multideterminant *ansatz* constructed from multiconfigurational self-consistent field theory (MCSCF) calculations achieves the MAD of 1.2 kcal/mol. Morales et al.^48^ were able to achieve subchemical accuracy (MAD of 0.8 kcal/mol) in atomization energies of the G2 set. In another study, chemical accuracy (and almost exact energies) could be achieved for the ionization potentials for several atoms from Li to Mg using full-configuration interaction.^49^ Yao et al. used the recently developed semistochastic heat-bath configuration interaction method to obtain excellent atomization energies for the G2 set with an MAD of 0.46 kcal/mol.^50^ Scemama et al.^51^ have recently developed a way to combine short-range XC functionals (from DFT) with a selected configuration interaction, which led to trial WFs with lower FN energies with compact multideterminant trial wave functions. The least MAD obtained reached 2.06 kcal/mol. Thus, the MADs obtained with the multideterminant approach are compatible with or better than the value obtained using the JsAGPs *ansatz* in this study.

The advantage of the multideterminant approach is that one can systematically improve the accuracy by increasing the number of SDs, and in theory, it can describe any ground state exactly with a sufficient number of SDs.^52^ However, the number of SDs that should be considered scales exponentially as the system size increases, which makes the approach computationally demanding for larger systems. While, in recent years, there have been successful efforts to reduce the number of determinants required for accurate calculations by truncating less important determinants,^53,54^ establishing a technique to apply the multideterminant *ansatz* to large systems with high accuracy is still an active field of research.^51,55^ Instead, the single-determinant approach we employed in this study enables the *ansatz* to have a large variational freedom, while keeping the computational cost lower, which allows one to tackle large molecules.^52^ It is an advantage of our single-determinant approach from the practical viewpoint. However, it is worth mentioning that a flexible single-determinant *ansatz* like the AGPs might suffer from its own limitations. For instance, the *ansatz* cannot be improved systematically unlike in the multideterminant approach. Thus, one should devise an appropriate *ansatz* from the physics viewpoint. For instance, Claudio et al.^52,56^ demonstrated that the atomization energy of the carbon dimer, for which spin fluctuations should be considered, is significantly improved by using the Pfaffian *ansatz*.^25^ Another limitation is that the optimization of a large number of nonlinear variational parameters in the single determinant *ansatz* should be handled efficiently. More specifically, due to the large number of variational parameters contained in the single determinant *ansatz*, the optimization may fall into physically incorrect local minima, or the optimization itself may diverge. To prevent them, in practice, one needs to prepare a good initial estimate and/or reduce the number of variational parameters by applying constraints in a physically meaningful way, but there are no general guidelines for these. Thus, applying QMC methods to large-scale systems beyond the fixed-node obtained by DFT or HF still requires developing new techniques.

## 5 Conclusions

To conclude, we have demonstrated the effectiveness of the AGPs *ansatz* (built from electron pairing functions or geminals) in VMC and LRDMC calculations using the TURBORVB QMC package. Using AGPs *ansatz* with the cc-pVTZ basis set, the LRDMC calculations for atoms recovered 95.6% correlation energy. The atomization energies computed using AGPs had an MAD of ∼1.6 kcal/mol from the experimental values. Chemical accuracy was achieved for several molecules, and the error was within ±5 kcal/mol for almost all the molecules. These results are quite encouraging as they show that allowing more variational freedom by utilizing a more flexible *ansatz* is a viable path toward more accurate QMC calculations. We believe that our work represents an important step toward showing that a combination of more flexible single determinant *ansatz* (JsAGPs) and nodal surface optimization can match the accuracy of quantum chemistry calculations, with the added advantages of excellent scaling and lower computational cost.

## Data Availability

TURBORVB is available from the web site [https://turborvb.sissa.it] upon request.

## Supporting Information Available

Computed total energies and references for molecular geometries (PDF)

## Dedication

Finally, we dedicate this paper to the memory of Prof. Sandro Sorella (SISSA), who passed away during this collaboration. We remember him as one of the most influential contributors to the QMC community in the past century, and in particular for deeply inspiring this work with the development of the ab initio QMC code, TURBORVB.
