# Supplementary material for: Toward Chemical Accuracy Using the Jastrow Correlated Antisymmetrized Geminal Power Ansatz
Source: J Chem Theory Comput. 2023 Apr 4;19(8):2222–9. doi: 10.1021/acs.jctc.2c01141 (PMC10134432; doi:10.1021/acs.jctc.2c01141)
Supplement: Supplementary file 1 — ct2c01141_si_001.pdf [file ct2c01141_si_001.pdf]

Supplementary Information

1 Computed total energies using various *ansatz*

Total energies of atoms and molecules computed using various *ansatz* are listed in the tables below. For, molecules the geometry references are also provided.

Table S1: Total energies [Ha] for atoms

Element	DMC-JsAGPs	DMC-JDFT	VMC-JsAGPs	DMC-JHF$^+$	Est. exact*
Li	-7.47815(7)	-7.4779(2)	-7.47777(6)	-7.47802(6)	-7.4781
C	-37.8371(2)	-37.8305(3)	-37.8255(2)	-37.8302(3)	-37.8450
N	-54.5785(4)	-54.5765(3)	-54.5614(1)	-54.5766(2)	-54.5892
O	-75.0550(3)	-75.0521(2)	-75.0331(2)	-75.0525(2)	-75.0673
F	-99.7199(3)	-99.7179(2)	-99.6920(2)	-99.7181(2)	-99.7339
Na	-162.2424(3)	-162.2415(3)	-162.2146(2)	-162.2397(2)	-162.2546
Si	-289.3344(3)	-289.3293(3)	-289.2817(2)	-289.3276(4)	-289.3590
P	-341.2262(3)	-341.2251(3)	-341.1740(2)	-341.2225(6)	-341.2590
S	-398.0709(3)	-398.0653(9)	-398.0065(2)	-398.0659(4)	-398.1100
Cl	-460.1050(2)	-460.0999(4)	-460.0365(2)	-460.0949(4)	-460.1480

Table S2: Total energies [Ha] for molecules

species_list	Geometry	DMC-JsAGPs	DMC-JDFT	VMC-JsAGPs	DMC-JHF$^+$	Est. exact*
LiH	a	-8.0699(3)	-8.070(1)	-8.06945(7)	-8.0704(2)	-8.070529
BeH	a	-15.2467(5)	-15.2454(2)	-15.2445(2)	-15.2460(2)	-15.246761
Li_2	a	-14.9937(3)	-14.9905(7)	-14.9932(2)	-14.9917(2)	-14.995084
CH	a	-38.4724(3)	-38.4632(3)	-38.4603(3)	-38.4638(3)	-38.478863
CH_2_singlet	a	-39.1280(3)	-39.1173(4)	-39.11534(7)	-39.1189(3)	-39.133920
CH_2_triplet	a	-39.1420(3)	-39.1401(4)	-39.1271(2)	-39.1413(2)	-39.149059
NH	a	-55.2104(3)	-55.2078(2)	-55.1929(1)	-55.2085(3)	-55.222744
CH_3	a	-39.8276(3)	-39.8273(3)	-39.8101(1)	-39.8277(2)	-39.835829
NH_2	a	-55.8698(3)	-55.8652(2)	-55.8517(1)	-55.8671(3)	-55.879554
OH	a	-75.7248(1)	-75.7204(5)	-75.7033(2)	-75.7222(3)	-75.737497
CH_4	a	-40.5086(2)	-40.5072(4)	-40.49311(8)	-40.5075(3)	-40.515269
H_2O	a	-76.4270(2)	-76.4225(5)	-76.4052(2)	-76.4240(3)	-76.439167
HF	a	-100.4478(3)	-100.4422(5)	-100.4230(2)	-100.4439(3)	-100.459713
NH_3	a	-56.5539(2)	-56.5495(4)	-56.53521(9)	-56.5525(3)	-56.564731
LiF	a	-107.4214(3)	-107.4187(3)	-107.3991(2)	-107.4187(2)	-107.434307
CN	b	-92.6950(3)	-92.6881(3)	-92.6556(1)	-92.6891(4)	-92.722961
C_2H_2	a	-77.3175(3)	-77.3137(3)	-77.2862(2)	-77.3137(4)	-77.332381
CO	b	-113.3038(3)	-113.2937(3)	-113.2676(2)	-113.2930(4)	-113.326318
HCN	a	-93.4107(3)	-93.4023(3)	-93.3777(2)	-93.4048(4)	-93.431085
N_2	b	-109.5165(3)	-109.5068(3)	-109.4702(5)	-109.5065(4)	-109.542697
HCO	a	-113.8342(3)	-113.8272(4)	-113.7954(2)	-113.8278(4)	-113.856915
NO	b	-129.8696(3)	-129.8608(3)	-129.82208(8)	-129.8612(4)	-129.900576
C_2H_4	a	-78.5735(2)	-78.5676(4)	-78.5427(2)	-78.5672(4)	-78.588951
H_2CO	a	-114.4876(3)	-114.4802(3)	-114.4509(2)	-114.4803(4)	-114.509104
O_2	b	-150.2949(3)	-150.2874(4)	-150.2483(2)	-150.2898(4)	-150.327203
C_2H_6	a	-79.8120(1)	-79.8107(2)	-79.7818(2)	-79.8098(5)	-79.826875
F_2	a	-199.4989(3)	-199.4852(8)	-199.4449(2)	-199.4868(4)	-199.530110
H_2O_2	a	-151.53355(9)	-151.5257(2)	-151.4846(2)	-151.5274(4)	-151.564235
H_4CO	a	-115.7081(2)	-115.7058(4)	-115.6710(2)	-115.7066(4)	-115.730774
N_2H_4	a	-111.8531(3)	-111.8488(5)	-111.8146(2)	-111.8502(5)	-111.877672
CO_2	b	-188.5658(3)	-188.5580(3)	-188.4982(2)	-188.5537(6)	-188.601471
SiH_2_singlet	c	-290.5836(4)	-290.5739(5)	-290.5372(2)	-290.5727(4)	-290.601705
SiH_2_triplet	a	-290.5490(3)	-290.5438(2)	-290.50460(9)	-290.5433(4)	-290.568877
PH_2	c	-342.4759(4)	-342.4674(3)	-342.4233(2)	-342.4676(5)	-342.503299
SiH_3	a	-291.2015(4)	-291.1933(3)	-291.1556(2)	-291.1947(4)	-291.222022
H_2S	a	-399.3620(2)	-399.3596(3)	-399.2930(2)	-399.3575(4)	-399.402426
HCl	a	-460.7743(2)	-460.7673(2)	-460.7028(2)	-460.7639(5)	-460.819376
PH_3	a	-343.1141(3)	-343.1098(2)	-343.0539(2)	-343.1068(5)	-343.147839
SiH_4	a	-291.8530(3)	-291.8518(3)	-291.7936(2)	-291.8506(5)	-291.873733
CS	a	-436.1801(3)	-436.1656(2)	-436.1024(2)	-436.1620(6)	-436.228940

Continued on next page

Table S2: Total energies [Ha] for molecules

species_list	Geometry	DMC-JsAGPs	DMC-JDFT	VMC-JsAGPs	DMC-JHF[+]	Est. exact[*]
SiO	a	-364.6950(3)	-364.6811(4)	-364.6225(2)	-364.6822(5)	-364.733068
SO	b	-473.3246(8)	-473.3113(3)	-473.2348(2)	-473.3126(6)	-473.378253
ClO	b	-535.2580(4)	-535.2449(4)	-535.1553(2)	-535.2422(6)	-535.320350
CH_3Cl	a	-500.0746(5)	-500.0636(4)	-499.9848(2)	-500.0618(6)	-500.123907
ClF	a	-559.9217(3)	-559.9080(5)	-559.8215(2)	-559.9057(6)	-559.982138
H_4CS	a	-438.6654(3)	-438.6584(4)	-438.5790(2)	-438.6556(6)	-438.711801
HOCl	a	-535.9203(3)	-535.9079(4)	-535.8239(2)	-535.9070(6)	-535.979997
SO_2	a	-548.5908(3)	-548.5752(4)	-548.4945(2)	-548.5704(7)	-548.658618
Na_2	c	-324.5103(4)	-324.5066(6)	-324.4556(2)	-324.5032(4)	-324.536291
NaCl	a	-622.5069(4)	-622.4977(5)	-622.4105(2)	-622.4913(6)	-622.560207
Si_2	a	-578.7899(3)	-578.7805(3)	-578.6889(2)	-578.7735(6)	-578.838636
P_2	a	-682.6364(4)	-682.6247(3)	-682.5193(2)	-682.6191(7)	-682.704451
S_2	a	-796.3076(4)	-796.2935(3)	-796.1822(2)	-796.2869(8)	-796.384237
Cl_2	a	-920.3023(3)	-920.2878(6)	-920.1569(2)	-920.2803(6)	-920.389991
Si_2H_6	c	-582.5298(5)	-582.5160(8)	-582.4238(2)	-582.5134(8)	-582.566752

[+]Nemec *et al.*, J. Chem. Phys. 2010, 132, 034111
[*]Bytautas *et al.*, J. Chem. Phys. 2005, 122, 154110
[a]Curtiss *et al.*, J. Chem. Phys. 1997, 106, 1063–1079
[b]Feller *et al.*, J. Chem. Phys. 2008, 129, 204105
[c]O'eill *et al.*, Mol. Phys. 2005, 103, 763-766
